# Insect Cecropins, Antimicrobial Peptides with Potential Therapeutic Applications

**DOI:** 10.3390/ijms20235862

**Published:** 2019-11-22

**Authors:** Daniel Brady, Alessandro Grapputo, Ottavia Romoli, Federica Sandrelli

**Affiliations:** 1 Department of Biology, University of Padova, via U. Bassi 58/B, 35131 Padova, Italy; daniel.brady@studenti.unipd.it (D.B.); alessandro.grapputo@unipd.it (A.G.); oromoli@pasteur-cayenne.fr (O.R.); 2 Institut Pasteur de la Guyane, 23 Avenue Pasteur, 97306 Cayenne, French Guiana, France

**Keywords:** antimicrobial peptides, insects, Cecropins, Cec-analogs, MDR infectious diseases

## Abstract

The alarming escalation of infectious diseases resistant to conventional antibiotics requires urgent global actions, including the development of new therapeutics. Antimicrobial peptides (AMPs) represent potential alternatives in the treatment of multi-drug resistant (MDR) infections. Here, we focus on Cecropins (Cecs), a group of naturally occurring AMPs in insects, and on synthetic Cec-analogs. We describe their action mechanisms and antimicrobial activity against MDR bacteria and other pathogens. We report several data suggesting that Cec and Cec-analog peptides are promising antibacterial therapeutic candidates, including their low toxicity against mammalian cells, and anti-inflammatory activity. We highlight limitations linked to the use of peptides as therapeutics and discuss methods overcoming these constraints, particularly regarding the introduction of nanotechnologies. New formulations based on natural Cecs would allow the development of drugs active against Gram-negative bacteria, and those based on Cec-analogs would give rise to therapeutics effective against both Gram-positive and Gram-negative pathogens. Cecs and Cec-analogs might be also employed to coat biomaterials for medical devices as an approach to prevent biomaterial-associated infections. The cost of large-scale production is discussed in comparison with the economic and social burden resulting from the progressive diffusion of MDR infectious diseases.

## 1. Introduction

The spread of infectious diseases resistant to conventional treatments has become an alarming phenomenon worldwide, prompting the United Nations and international agencies to call for immediate and coordinated actions to avoid a possible global drug-resistance crisis [1]. Drug-resistance phenomena involve not only antibacterial compounds, but also antiviral, antifungal, and antiprotozoal therapeutics in all countries, independent of their economic level. Currently, estimates indicate that drug-resistance cases result in 700,000 deaths per year worldwide, and without direct action, annual death tolls could reach 10 million by 2050 [1]. Research and development of new therapeutics have been included at the forefront of the proposed actions to tackle the global antimicrobial resistance phenomenon [1]. Several lines of evidence indicate that the utilization of antimicrobial peptides (AMPs) represents a compelling option [2,3].

AMPs are naturally occurring peptides produced as a first line of defense against pathogenic infections by virtually all living species, from bacteria to mammals [2]. AMPs play an essential role in those organisms that lack an adaptive immune system and base their defense only on the innate immune response, such as invertebrates. Of these, Insecta is the largest animal class on Earth, containing 50% of all known animal species, and represents a wide source of AMPs. To date, 305 out of the 3087 AMPs listed in the Antimicrobial Peptide Database (APD; Available online: http://aps.unmc.edu/AP [4]) are derived from insects. Notwithstanding, these numbers are likely to increase extensively given the current growth of accessible genomic, transcriptomic, and proteomic insect datasets, which will accelerate the identification of new putative AMPs available for subsequent analyses and characterization.

First identified about 40 years ago, a wide variety of insect AMPs has since been characterized. These molecules have been intensively studied, not only for their physiological role in insect immunity, but also as potential alternatives to conventional antibiotics in the treatment of infectious diseases [5,6,7]. Moreover, some insect AMPs have been shown to possess immunomodulatory functions as well as anticancer activity [5,6]. These biological properties, combined with modern advances in biotechnology, have resulted in a renewed interest in insect AMPs and their potential to combat modern biomedical challenges.

Insect AMPs can be classified on the basis of their sequence and structure into three groups: (i) α-helical peptides, lacking in cysteine residues (e.g., Cecropins (Cecs) and Moricins); (ii) β-sheet cysteine-rich peptides (e.g., Defensins and Drosomycins); and (iii) linear-extended peptides, often characterized by high proportions of peculiar amino acids (aa) such as proline, arginine, tryptophan, glycine, and histidine. Both proline-rich peptides (e.g., Apidaecins, Drosocins, and Lebocins) and glycine-rich AMPs (e.g., Attacins and Gloverins) belong to this group. As the different classes of insect AMPs have been recently reviewed in [5,6,7], here we focus on Cecs, one of the largest groups of insect AMPs. We report a comprehensive overview of the Cec family in insects, and provide up-to-date models explaining their mode of action. We then highlight the antimicrobial, anti-inflammatory, and antitumor activities of natural Cecs and Cec-like peptides, as well as of synthetic Cec-analogs, which carry different types of sequence modifications. The potential benefits and limitations in the development of Cec-based antibacterial therapeutics are also presented.

## 2. The Family of Cecropins in Insects

Cecs and other Cec-like peptides, including Sarcotoxins, Stomoxins, Papiliocin, Enbocins, and Spodopsins, form the most abundant family of linear α-helical AMPs in insects (Table 1). Cec AMPs were first isolated from the hemolymph (insect blood) of the lepidopteran *Hyalophora cecropia* and were characterized for their antimicrobial activity against several Gram-positive and negative bacteria [8,9,10]. Subsequently, these peptides have been identified in two other orders of Hexapoda, Coleoptera and Diptera, as well as in other species of Lepidoptera [7,11]. In evaluating several genomes, the identification of Cec and Cec-like peptide sequences was not successful in other insect orders ([11]; this review), including Hymenoptera, which is considered the sister clade of the other holometabolous insects [12]. However, Cecs have been identified in other animals, such as Styelin in tunicates [13], and Cec P1, first isolated from pigs [14], but then found to belong to the Nematode *Ascaris suum* [15]. Cec-like peptides have been also identified in the bacterium *Helicobacter pylori* [16]. Since these peptides derive from the N-terminal part of ribosomal protein L1 (RpL1) and are similar to Cecs from *H. cecropia*, Pütsep and colleagues suggested that Cecs may have evolved from an early prokaryote RpL1 gene [16]. Indeed, the homology of Cecs has been debated and some authors consider them a single family, with Dermasptin (amphibians), Ceratotoxin (insects), and Pleurocidin (fish) forming a Cec superfamily [17]. Indeed, the members of the Cec family show sequence similarity that enabled the identification of a first sequence signature of Cecs from some species of Brachycera (Diptera) and Lepidoptera (i.e., [KR]-[KRE]-[LI]-[ED]-[RKGH]-[IVMA]-[GV]-[QRK]-[NHQR]-[IVT]-[RK]-[DN]-[GAS]-[LIVSAT][LIVE]-[RKQS]-[ATGV]-[GALIV]-[PAG]) [17]. This has been updated to include Culicomorpha (mosquitoes) and nematodes (i.e., [KRDEN]-[KRED]-[LIVMR]-[ED]-[RKGHN]-X(0,1)[IVMALT]-[GVIK]-[QRKHA]-[NHQRK]-[IVTA][RKFAS]-[DNQKE]-[GASV]-[LIVSATG][LIVEAQKG]-[RKQSGIL]-[ATGVSFIY]-[GALIVQN]) [18]. However, the lower similarity between Cecs from insects and other organisms and the lack of Cec-like peptides outside the clade of Coleoptera, Diptera, and Lepidoptera prompted other authors to suggest that insect *Cec* genes may have evolved just once in the common ancestor of these holometabolous orders, implying that insect and non-insect Cecs are not homologous [11].

Although most Cec diversity is found in insect taxa with whole genome sequences, phylogenetic analysis suggests that there is a significant undiscovered diversity in other holometabolous insects. Within different species, *Cec* genes are generally present in a variable number of copies organized in clusters or dispersed in the genome and can include both functional and non-functional elements (pseudogenes). For example, among Diptera, *Drosophila melanogaster* shows four functional genes (*Cec A1*, *A2*, *B*, and *C*) and two pseudogenes (Cec *ψ1* and *Cec ψ2*), clustered in a ~7-kb region [68,69]; to date, *Musca domestica* displays the largest gene family, characterized by 12 *Cec* members [70]. Among Lepidoptera, the *H. cecropia Cec* locus spans ~20 kb and contains three *Cec* genes (A, B, and D) [9,71], coding for three Cec A, B, and D functional peptides. Moreover, *H. cecropia* shows the additional Cec forms C, E, and F, that have been isolated in low amounts, and classified as allelic variants or degradation products of the three main A, B, and D forms [9]. In the domesticated silkworm *Bombyx mori*, the *Cec* gene family is composed of at least 14 elements (two *Cec A* (*A1* and *A2*), six *Cec B* (*B1*–*B6*), one *Cec C*, two *Cec D* (*D* and *D2*), one *Cec E*, and two *enbocins* (*enb* 1 and 2)), organized in two clusters, mapping on two different chromosomes [72]. In Coleoptera, functional *Cec* genes have been identified in species like *Acalolepta luxuriosa* (Cec; [20]), *Oxysternon conspicillatum* (Oxysterlins; [19]), and *Paederus dermatitis* (Sarcotoxin Pd; [21]), whereas only non-functional *Cec* pseudogenes have been reported in the coleopteran model *Tribolium castaneum* [73,74].

Phylogenetic analyses and single genome sequencing revealed that insect Cec and Cec-like peptides originated via gene duplication and evolved via a birth and death model of gene evolution [72,75]. The occurrence of gene duplication events is confirmed by the presence of transposable elements in both 5’ and 3’ flanking regions, and repeated gene duplication within species. Furthermore, tandem gene arrangement within the genome, non-functionalization, and loss of some *Cec* gene copies, and the presence of highly divergent and highly similar gene copies within species all support the gene duplication hypothesis [75,76]. Compared to other AMPs, Cecs show no sites under positive selection [77,78], but frequent duplication events may be adaptive, enabling new gene copies to mutate and acquire novel antimicrobial properties [79].

**Figure 1:**
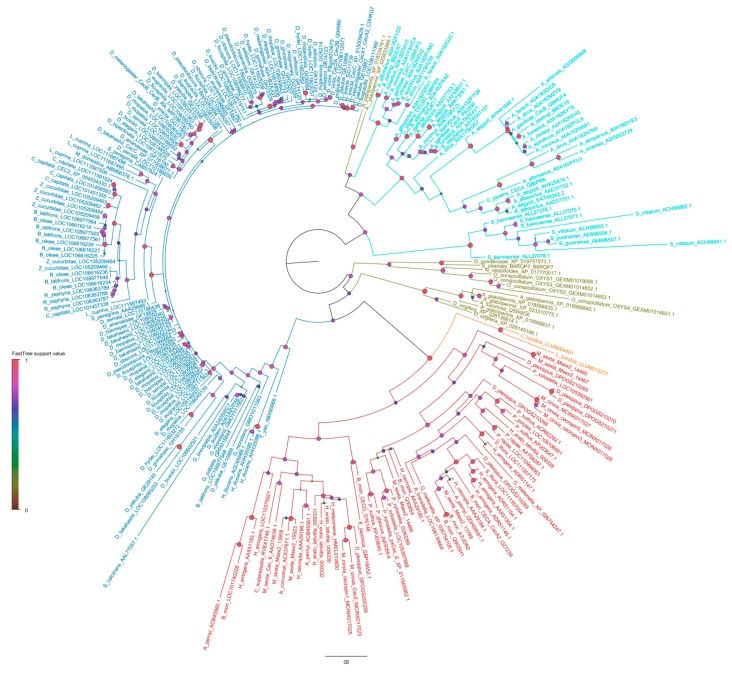
Phylogenetic tree of insect Cecropins (Cecs) and Cec-like peptides. Maximum likelihood mid-point rooted phylogenetic tree showing the relationships of insect Cecs and Cec-like peptides. The tree was obtained with FastTree 2.1.5 software with the WAG + Γ model [81]. Lepidoptera peptides are shown in red, Trichoptera in orange, Diptera Brachycera in dark blue, Diptera Culicomorpha in light blue, and Coleoptera in green. Full-length Cecs and Cec-like peptides were downloaded from the OrthoDB database (Available online: https://www.orthodb.org/), which contains 230 sequences in 61 species of Lepidoptera and Diptera. Other sequences, including those from *Simulium*, Trichoptera, and Coleoptera, were downloaded from NCBI and UniprotKB. Sequences were aligned with ClustalW using default parameters in Geneious 8.1.9 (BioMatters). Identical sequences within species were removed leaving a total of 254 Cecs and Cec-like peptides. Either UniprotKB or NCBI accession number are reported for each sequence in the tree. Circle at the nodes indicate node support obtained with Shimodaira–Hasegawa-like local support. Shade (as shown in the legend) and circle size are proportional to the node support value (0–1). The scale bar corresponds to estimated amino acid substitutions per site.

Phylogenetic analysis (Figure 1) [11,72,76] shows that Cecs from Lepidoptera form a monophyletic group (derived from a single ancestral gene) and evolved independently in this order of insects [22]. In contrast, the phylogenetic relationships of Cecs from Diptera and Coleoptera are more complex. Complementing previous phylogenetic analyses [76,80], we included new data from mosquitos and several Coleoptera species. Cecs from Diptera and Coleoptera are both paraphyletic, suggesting that Cecs originated before these lineages diverged. Within Diptera, Cecs from Brachycera (which include *Drosophila*) form a monophyletic group, which is closely related to that of Lepidoptera and is distinct from that of Culicomorpha (mosquitos) (Figure 1).

## 3. Cec Gene Expression and Mechanism of Action Against Microorganisms

**Figure 2:**
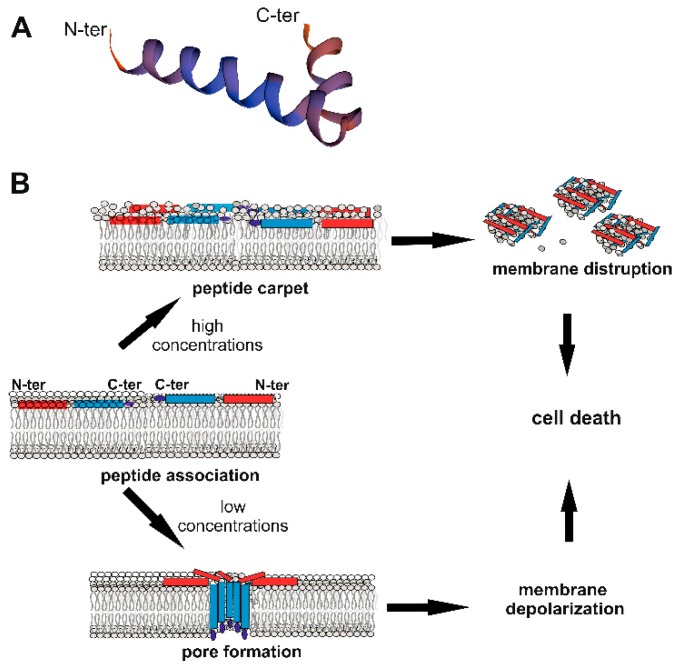
Cecropin (Cec) structure and mechanisms of action against bacteria. (**A**) Structure of the mature 35 aa *B. mori* Q53 Cec B natural variant [53] obtained using SWISS-MODEL (Available online: https://swissmodel.expasy.org/), showing N- and C-terminal α-helices linked through a flexible hinge region. (**B**) Model of action against bacteria. Cecs associate with the bacterial membrane, with the long axes of the α−helical domains parallel to the lipid bilayer surface. Polar residues interact with the lipid phosphates; non-polar residues bury in the hydrophobic core of the membrane. At high concentrations (upper part), Cecs form a carpet-like structure with detergent-like properties, disrupting membranes. At lower concentrations (lower part), Cecs form pores, which affect the cellular electrolyte balance, causing bacterial death [85]. The pore is formed of different Cec molecules organized as oligomers, with C-terminal hydrophobic domains submerged into the phospholipidic hydrophobic chains [86]. The red rectangle represents the N-terminal helix, the blue one the C-terminal helix; the dark blue ellipse indicates the C-terminal amidated residue.

In the absence of any infections, *Cec* genes can be constitutively expressed at low levels in different body compartments, as demonstrated in the *Drosophila* reproductive tract [82] or in the silkworm *B. mori* midgut or fat body (a structure equivalent to the mammalian liver) [83]. Following an immune challenge, *Cecs* become highly transcribed in several tissues, such as gut epithelia or epidermis during local infections, and the fat body and hemocytes, during systemic infections (e.g., [51,82,83]). Like other AMPs, Cecs are translated as immature pre-peptides, undergo proteolytic cleavage of the N-terminal signal peptide, and are secreted in a mature and active form [5,7]. Before maturation, Cec sizes range between 58 and 79 aa, while active forms contain between 34 and 55 residues (Table 1). Experimental and computational analyses indicated that Cec and Cec-like peptides are structurally related and are characterized by an N-terminal basic, amphipathic domain linked to a more hydrophobic C-terminal segment, through a flexible proline- and glycine-rich hinge region (Figure 2A; [5,7,84]).

Insect Cecs and Cec-like peptides are generally active against Gram-negative bacteria and to a lesser extent, Gram-positive bacteria (Table 1). Some have been demonstrated to also exhibit antifungal activity (Table 1). Moreover, Cec and Cec-like peptides were shown to have a low toxicity against normal mammalian cells and a weak or absent hemolytic effect against mammalian erythrocytes (Table 1). As for other cationic AMPs, the ability of these peptides to target microorganisms without interacting with host eukaryotic cells relies on the difference in composition of the respective cell membranes. Bacterial membranes are predominantly composed of negatively charged compounds (e.g., phosphatidylglycerol, cardiolipin, and phosphatidylserine), while eukaryotic membranes are positively charged by the presence of zwitterionic phospholipids and cholesterol [87]. Furthermore, Gram-negative bacteria possess an external membrane rich in negatively charged Lipopolysaccharides (LPS, also known as endotoxin), whereas in Gram-positive bacteria, the peptidoglycan is anchored to the cytoplasmic membrane by negatively charged teichoic acids. It is also generally thought that the discrimination between fungi and other eukaryotic host membranes is due to the different sterol compositions of their respective membranes [87].

Using chemically synthetized natural Cec variants and modified analogs, several studies have been performed to explain the Cec action mechanism against pathogens, as well as to identify the functions of specific residues within the peptide. Most mature Cec peptides contain a tryptophan residue in the first or second positions, which is considered important in conferring full antimicrobial activity to the peptide [5,7,84,88]. A study performed on Papiliocin, from the lepidopteran *Papilio xuthus*, suggested that the presence of tryptophan^2^ and phenylalanine^5^ aromatic residues in the N-terminal region are essential for the full-length peptide to interact with LPS in the outer membrane, and permeabilize the inner membrane of Gram-negative bacteria [58]. However, some dipteran Cecs, such as those from the black fly *Simulium bannaense* and the mosquito *Aedes aegypti* have been shown to be highly effective against different bacteria, although lacking an N-terminal tryptophan residue [22,25].

In several cases, Cec peptides undergo amidation of the C-terminal residue, a post-translational modification, which increases both antimicrobial activity and the action spectrum of the peptide [6,7]. It has been demonstrated that the antimicrobial activity of Cec AMPs relies on the structure they assume in the presence of bacterial cells. Circular dichroism analyses showed that in aqueous solution, Cecs have a random coiled structure but adopt α-helical conformations upon interaction with microbial membranes, where they exert a lytic effect [53,58,84,86]. Although some aspects remain unclear, it is currently accepted that Cec peptides do not interact with specific receptors but initially associate with the bacterial membrane along the axes of the α-helical domains parallel to the lipid bilayer surface. At this level, the polar residues of the peptide interact with the lipid phosphates, while the non-polar side chains burrow in the hydrophobic core of the membrane [84] (Figure 2B). In a first model of action, the continuous accumulation of peptides at the bacterial lipid bilayer leads to the formation of a peptide “carpet” on the membrane surface. This “carpet” structure possesses intrinsic detergent-like lytic properties, which disintegrate the membranes [84]. Cec P1 [14,15] and *H. cecropia* Cecs, when administrated at high concentrations (Cec P1 > 25 μM; *H. cecropia* Cecs > 5 μM), appear to act through this carpet-like mechanism (Figure 2B) [84,85]. However, at lower concentrations (2–5 µM)*, H. cecropia* Cecs are able to associate with membranes and form channels or pores, which affect cellular electrolyte balance and in turn cause the death of the microorganism (Figure 2B) [84,85,86]. Initially, it was postulated that the N-terminal amphipathic regions of the peptides were involved in the formation of the pore (called “type II channel”), with the positively charged residues forming the inner channel [89,90]. Subsequent authors have hypothesized that the C-terminal hydrophobic domains of the peptides insert into the membrane giving rise to a more stable pore (type I channel), in which the polar aa of the C-terminal helices are oriented toward the center of the pore [85,86,90]. Efimova and colleagues analyzed the effect of *H. cecropia* Cecs A and B in model lipid membranes, with or without small molecules capable of modifying the membrane physical-chemical properties [85,86]. Using these data, they developed a model in which Cec peptides first interact as monomers with the hydrophilic heads of the lipid bilayer surface, acting parallel to the membrane plane. Next, the peptides submerge their C-terminal hydrophobic domains into the phospholipidic hydrophobic chain. Individual Cec molecules then organize into oligomers forming ion-permeable pores in the cell membrane (Figure 2B). Other monomers can then insert into the pores, increasing the ion channels’ conductance. The authors also postulated that all the steps of this process are reversible and in equilibrium [86]. This pore model therefore resembles the “barrel-stave” model, in which the different C-terminal regions of the *H. cecropia* Cec peptides are organized to form a barrel penetrating the bacterial membrane. However, in cases where the peptide is shorter than ~ 22 aa (e.g., synthetically Cec-derived analogs, see below), the structure of the pore might be more similar to the so-called “toroidal-pore” model, in which the pore is composed by both peptides and lipids [84].

As mentioned above, natural Cec and Cec-like peptides show a higher activity against Gram-negative compared to Gram-positive bacteria. This feature has been related to the difference in the intrinsic properties of bacterial membranes (i.e., lipid composition, charge density, and electrochemical potential across the membrane), as demonstrated when evaluating *H. cecropia* Cec B against protoplasts obtained from Gram-negative *Escherichia coli* and Gram-positive *Staphylococcus aureus* or *S. epidermidis* [46]. Moreover, a recent study on natural Papiliocin and its modified derivatives associated the Cec’s preferential activity against Gram-negative bacteria specifically with the presence of the C-terminal helix. In fact, compared to the full-length natural form, a truncated Papiliocin carrying only the N-terminal portion was less effective against Gram-negative, and more active against Gram-positive bacteria [58].

Finally, in a study evaluating the interaction between different *B. mori* natural Cec B variants and live Gram-negative *Pseudomonas aeruginosa*, it was suggested that Cecs might first affect the outer bacterial membrane, enabling the translocation of the peptide to the inner membrane, resulting in the disorganization of both lipid bilayers [53].

## 4. In Vitro Antimicrobial Activity of Natural Cecs and Synthetic Cec-Analogs

Numerous basic research studies have shown that natural Cecs or synthetic Cec-analogs can have antibacterial, antifungal, antiviral, and antiprotozoal properties (Table 1 and Table 2 and reference herein). Although there is a lack of uniformity among these studies, the peptides have generally exhibited a high in vitro activity against Gram-negative bacteria. These also included multidrug resistance (MDR) strains listed by the World Health Organization (WHO) in the three “critical, high and medium” priority groups, requiring the development of new antibiotics [91].

Cec peptides were effective against laboratory strains of *P. aeruginosa* and different *Enterobacteriacae* spp. (including *K. pneumoniae* and *E. coli*), bacterial species belonging to the WHO first critical group. *M. domestica* Mdc, black fly SibaCec, and dung beetle Oxysterlins were active against MDR and clinically isolated *E. coli* strains [19,33,108], while *H. cecropia* Cec A and *P. xuthus* Papiliocin efficiently killed MDR *P. aeruginosa* isolates [45,58]. Mdc and SibaCec were also active against reference strains belonging to *Acinetobacter baumanii*, also critical on the WHO list [22,35]. Lepidopteran *H. cecropia* Cec A, *P. xuthus* Papiliocin, Cec D from the mosquito *A. aegypti*, and different synthetic CAM hybrids (formed from the fusion of the N-terminal regions of *H. cecropia* Cec A and *Apis mellifera* Mellitin) were effective against MDR *A. baumanii* strains [25,45,58,95].

Several natural Cecs and Cec-analogs have also shown activity against the food-borne Gram-negative pathogen *Salmonella typhimurium*, included in the high priority group of the WHO list (e.g., [19,23,25,58,80,98]). In addition, some dipteran Cec AMPs, such as those from the mosquitos *Aedes albopictus* and *Culex pipens*, were active against *Francisella novicida*, a facultative Gram-negative bacterium used as reference species to model *F. tularensis*, a zoonotic pathogen causing tularemia in humans and animals [28].

It is important to note that, although natural Cecs and Cec-like peptides generally demonstrated an antimicrobial activity against Gram-positive bacteria such as *Bacillus* spp and *Micrococcus luteus*, the vast majority were not or weakly active against *S. aureus*, which belongs to the high priority group on the WHO list (an exception appears to be the horse fly Cec TY1, which is reported to be more active against *S. aureus* than *E. coli*; [29]). Interestingly, synthetic Cec-analogs were active against *S. aureus*. In particular, an anti-*S. aureus* activity characterized CAM peptides [94,96,98,99], and other chimeric hybrids, such as CA-MA or CA-LL37, obtained from the fusion of *H. cecropia* Cec A N-terminal fragments with portions of *Xenopus laevis* Magainin [102] or human LL-37 AMP [106], respectively (Table 2). Similarly, ΔM2 (a synthetic variant of *Galleria melonella* Cec D with modified residues in the N-terminal region; [54]) and Cec XJ forms (2-aa longer variants of *B. mori* Cec B; [107]) were also effective against *S. aureus* (Table 2).

Moreover, Cec D from the lepidopteran *G. mellonella* showed antibacterial activity against *Listeria monocytogenes*, a Gram-positive bacterium causing listeriosis, a food-borne infection, which can cause meningitis, meningoencephalitis, and fatal sepsis [54,109].

Several natural Cecs and analog derivatives have also been tested against a variety of fungi (Table 1 and Table 2). Although the peptides were not all effective against these microorganisms, *H. cecropia* Cecs A and B [44], *P. xuthus* Papiliocin [58], *Artogeia rapae* Hinnavins [65,66], Cec A from the mosquito *Anopheles gambiae* [23], and a Cec-analog derived from the D-enantiomerization of *Antheraea pernyi* Cec B [93], were active against *Candida albicans*, an opportunistic pathogen responsible for candidiasis in human hosts [110]. Synthetic analogs also showed in vitro antiprotozoal activities, as demonstrated for SB-37 and Shiva, which were effective against *Trypanosoma cruzi* and *Plasmodium falciparum* [92], and a chimeric CAM hybrid active against *P. falciparum* [94] (Table 2). Finally, several Cec and Cec-analog peptides have also been tested for their potential antiviral activity (Table 1 and Table 2). *H. cecropia* Cec A was able to suppress replication of human immunodeficiency virus 1 (HIV) by inhibiting viral gene expression [43], while Cec D was active against the porcine reproductive and respiratory syndrome virus (PRRSV) [47]. Additionally, engineered CA-MA hybrids were shown to inhibit virus–cell fusion activity [104].

## 5. Anti-Inflammatory Properties of Natural Cecs and Synthetic Cec-Analogs

Some Cec AMPs have been explored for their potential anti-inflammatory activity. Inflammation is an organism-protective response against different factors, including pathogens, which contributes to the removal of harmful foreign agents and to the initiation of reparative processes. An uncontrolled inflammatory response can however be dangerous, eliciting different acute or chronic diseases (reviewed in [111]). During Gram-negative infections, the release of LPS can overstimulate the innate immune system resulting in septic shock [112]. Several Cecs and Cec-analogs are able to bind LPS and have shown both in vitro and in vivo anti-inflammatory properties. Specifically, peptides derived from Lepidoptera (*H. cecropia* CecA [45], Papiliocin and derivatives from *Papilio xuthus* [57,58,113], Cec B, and a synthetic analog from *A. pernyi* [49]) were able to inhibit the production of nitric oxide and the transcription of several pro-inflammatory genes in LPS-treated murine cells, in vitro. Similar properties characterized natural Cecs from Diptera, such as Cec TY from the horsefly *Tabanus yao* [108], SibaCec from the black fly *S. bannaense* [22], and AeaeCec 1 from the mosquito *A. aegypti* [26]. In addition, an in vivo study showed that an intraperitoneal administration of *H. cecropia* Cecs A and B or a Papiliocin analog were able to reduce bacterial concentrations, plasma endotoxin levels, and mortality in *E. coli*-infected rodent models [113,114]. Finally, *M. domestica* Mdc was shown to alleviate colonic mucosal barrier impairments induced in mice by a *Salmonella typhimurium* infection, with a reduction in the colonic inflammation and oxidative stress response [115]. These studies demonstrate the dual antimicrobial and anti-inflammatory functions of Cec AMPs, underpinning their potential utilization in biomedical applications.

## 6. Antitumor Activity of Natural Cecs and Synthetic Cec-Analogs

Although the antitumor activities of Cecs and Cec-analogs have been less widely studied than their antimicrobial activities, these peptides indeed possess antitumor properties. These characteristics, for example, refer to *H. cecropia* Cecs A and B, *M. domestica* Mdc, *B. mori* Cec XJ derivatives, and the chimeric CAM and CA-MA hybrids, which were active against different types of human and rodent cancer cell lines in vitro [80,100,116,117,118,119,120,121]. Cec XJ and Mdc were also shown to inhibit proliferation and promote apoptosis of transformed cells in vitro [80,120]. Interestingly, when tested at the same concentrations, none of the analyzed AMPs showed any cytotoxic effects against normal cell lines. This selective antitumor activity might in part depend on the variable membrane compositions and fluidity of transformed compared to non-transformed cells [122]. Finally, Cec antitumor activity was also demonstrated in in vivo mammalian models, as shown for the *H. cecropia* Cec B and *B. mori*-derived Cec XJ, both improving the survival of mice bearing malignant ascites [117,123], indicating the potential of these AMPs as anticancer therapeutics.

## 7. Health Benefits of Natural Cecs and Synthetic Cec-analogs: Future Potential and Limitations

Several studies have suggested that some natural Cecs and synthetic-derived Cec peptides represent promising molecules for the development of new antibacterial drugs. Resistance to conventional antibiotics is a global phenomenon, involving not only the health system, but also livestock production [124]. The potential of insect AMPs as antimicrobial dietary supplements has been recently reviewed [125]. In addition, different studies reported the use of transgenesis to produce Cec-overexpressing plants and animals exhibiting greater resistance to pathogenic infections compared to non-transformed controls (e.g., [126,127]). Although effective, the use of transgenic strategies is limited by the regulatory laws of different countries and is not discussed in detail in this review. In the following paragraphs, we consider the potential of peptides belonging to the Cec family as therapeutics for clinical applications.

### 7.1. Potential of Natural Cecs and Cec-analogs as Antibacterial Drugs

Unlike other AMPs, Cec and Cec-analog peptides have generally shown low in vitro toxicity, evaluated as cytotoxicity against normal mammalian cell lines and/or hemolytic activity against human or rodent erythrocytes (Table 1 and Table 2). Although there is variation among the analyzed Cecs, the peptide concentrations showing initial toxicity against mammalian cells were one or two orders of magnitude higher than the minimum inhibitory concentration (MIC) values against the analyzed bacteria. Interestingly, a low toxicity was also typical of different Cec chimeric hybrids, including some CAM, CA-MA, and CA-LL37 peptides [100,102,106], which generally showed a wide action spectrum against both Gram-positive and-negative bacteria (Table 2).

Several natural Cecs and synthetic derivatives have shown a high stability to heat treatments and/or pH variations (e.g., [53,98,107])**.** In addition, they usually maintained their antimicrobial activity in complex biological fluids, mimicked in vitro by using high concentrations of serum, as well as in the presence of elevated levels of divalent cations such as Ca^2+^ and Mg^2+^, which show 1–2 and 0.5 mM concentrations in human saliva, respectively, and might reduce or inhibit AMP effectiveness (e.g., [37,53,107,128]). Similarly, natural Cecs and Cec-analogs were also active when analyzed in the presence of high concentrations of Na^+^, typical of airway surface fluids from patients affected by cystic fibrosis, who often suffer lung infections from bacteria such as *P. aeruginosa*, *A. baumanni*, or *S. aureus* (e.g., [49,53,98,102,129]).

The vast majority of data on Cec antimicrobial activity is derived from in vitro analyses. However, some studies have shown the potential of these peptides in vivo. For example, single intraperitoneal administrations of *H. cecropia* Cecs A and B, and *Danaus plexipibus* DAN2 decreased mortality in acutely *E. coli*-infected rodent models [114,130]. In addition, mice subjected to DAN2 doses two-fold higher than their most effective antibacterial concentration did not display any behavioral or morphological abnormalities, demonstrating in vivo that these peptides lack toxic effects after acute treatments [130].

With the prospect of employing natural Cecs and Cec-analogs in the treatment of infectious diseases, one of the potential problems is the capability of pathogens to develop resistance to these AMPs. Antimicrobial resistance is a complex phenomenon involving the development of intrinsic and/or acquired factors able to inactivate a compound or modify a target, nullifying the action of the specific drug. Currently, most considerations about, and data on AMP resistance in the literature, refer to bacteria. Although it is generally accepted that bacteria do not develop resistance to AMPs as easily as to conventional antibiotics, cases of bacterial resistance have been reported for non-Cec AMPs [125,131]. However, a recent study on *E. coli* compared the bacterial mutation rate induced by treatments with antibiotics with those with cationic AMPs, including *H. cecropia* Cec A [132]. Unlike antibiotics, none of the analyzed AMPs increased *E. coli* mutation rates. The authors linked this phenomenon to the inability of these AMPs to activate bacterial stress pathways that promote DNA mutagenesis [132]. Since the family of Cecs act against bacteria with a similar bactericidal mechanism at a molecular/cellular level, these data suggest that these AMPs are unlikely to stimulate the development of new intrinsic resistance factors linked to a mutation rate increment, at least in the *E. coli* model.

Long-term exposure to low levels of an antimicrobial compound is an important driver of antimicrobial resistance. Promising data have shown that following long-term treatments with the hybrid CAM peptide at sub-lethal concentrations did not significantly alter the peptide MIC. Following treatment, CAM remained effective against both laboratory reference and MDR *P. aeruginosa* strains, whereas similar serial exposures to sublethal doses of gentamicin or LL-37 increased their effective MICs on the same bacterial strains [97]. These studies provide important data suggesting that treatments with Cec and Cec-analog peptides do not easily induce antimicrobial resistance. However, dedicated studies analyzing all aspects of bacterial resistance, including the possible acquisition of exogenous factors through horizontal gene transfer, should be performed for each promising Cec or Cec-analog antibacterial candidate.

An innovative approach that is gaining interest is the use of AMPs as adjuvants in combination with conventional antibiotics [133]. Simultaneous treatments of AMPs and antibiotics can determine synergistic antimicrobial effects that are able to increase therapy efficacy and lower administration doses, in turn decreasing potential toxicity side effects. This aspect should be evaluated for each Cec or Cec-analog candidate, since the indications derived from in vitro studies performed on Cec-analog peptides, such as CAM or Cec-LL37 hybrids, showed variable synergistic activity grades, depending on the types of antibiotics and bacterial species (e.g., [96,97,106,134]).

### 7.2. Natural Cecs and Cec-Analogs as Anti-Biofilm Compounds

Biofilms are bacterial communities embedded in an extracellular matrix of polysaccharides, proteins, lipids, and DNA [135]. The bacteria forming biofilms display numerous interesting emergent social behaviors but are less susceptible to the effectors of the human defense system and exhibit a higher tolerance to conventional antibiotics, conferred in part from the extracellular matrix [135,136]. Several bacteria responsible for infections in hospitalized and/or immunodepressed patients can form biofilms, including Gram-negative *P. aeruginosa*, *A. baumanni*, *K. pneumoniae*, and Gram-positive *S. aureus*, and *S. epidermidis* [135]. Estimates indicate that biofilm infections are associated with at least two-thirds of all clinical infections [136]. In humans, many surfaces can be infected by biofilms, such as skin, teeth, ears, bones, and the respiratory and urinary tracts. Biofilms can also grow on medical devices, such as artificial implants, valves, and catheters, frequently used in modern medicine as feasible solutions to rescue compromised organs. Medical devices are composed of different types of biomaterials, and a great effort has been made to develop safe biomaterials. However, biomaterial microbial colonization remains one of the major problems related to the use of such devices. Contaminated devices can cause biomaterial-associated infections that are difficult to treat with conventional antibiotic therapies, triggering severe consequences for patient health [137].

Innovative anti-biofilm treatments are therefore needed [136,137] and Cecs and Cec-analogs might represent a promising solution. Two in vitro studies have demonstrated anti-biofilm effects of CAM hybrids, alone and in combination with conventional antibiotics, to treat both *P. aeruginosa* and *S. aureus* biofilms [134,138]. In addition, the use of AMPs to coat biomaterials during device manufacturing is considered a promising strategy to prevent biomaterial-associated infections (reviewed in [137]). Different studies have explored the possibility of using Cec and Cec-analog peptides in the functionalization of several types of materials used in biomedicine, such as hydrogels [139], polyurethane surfaces [140], as well as silk fibroin films or fibers [141,142]. These peptide-enriched materials were able to inhibit the growth of *E. coli* [139,141,142] and *S. epidermidis* [140], supporting the potential of Cec and Cec-analog peptides in these applications.

### 7.3. Biomedical Applications of Natural Cecs and Cec-Analogs: Limitations and Potential Solutions

All potential treatments that aim to inhibit pathogenic infections as well as combat antimicrobial resistance suffer limitations in their overall efficacy, including AMPs. Peptides are subject to degradation by naturally occurring proteases, such as trypsin, which is abundant in the digestive tract, and trypsin-Cec degradation has been demonstrated in *B. mori* (Cec B and Cec XJ variants, specifically) [53,107]. Furthermore, Cec peptides can also be targets for human elastase, which is produced by neutrophils, defense cells recruited during infections. In addition, Cec AMPs might be inactivated by proteases secreted by pathogens, such as *Pseudomonas* elastase and *S. aureus* V8 protease [98]. However, AMP sensitivity to proteolytic degradation can be limited in a number of ways. The substitution of specific residues is one such method to inhibit proteolytic degradation; this was recently demonstrated in CAM peptides, where a four-tryptophan-substitution variant (CAM-W) lost susceptibility to degradation by each of the enzymes mentioned above (Table 2) [98]. Peptide stability against proteolysis can also be achieved by the substitution of the natural L-residues with their respective D-enantiomers. This method was used to generate a whole D-enantiomer of the *A. pernyii* L-Cec B [93]. The obtained D-Cec B peptide maintained potent biocidal activity, while resisting the proteolytic activity that degraded the L-form (Table 2) [93].

In addition, enzymatic degradation might be limited by employing novel strategies based on the use of nanotechnologies. Indeed, the use of nanoparticles (NPs) to develop new formulations for AMP delivery is considered an improvement able to enhance peptide stability, while increasing peptide bioavailability and efficiency at the desired target site, as well as reducing the risk of possible toxic side effects [3,143]. In a recent study, Rai and colleagues demonstrated that the conjugation of CAM peptides to gold NPs enhanced in vitro CAM antimicrobial activity and stability as well as in vivo efficacy in a sepsis mouse model [144]. These encouraging results are opening new prospects for the use of Cec and Cec-analog peptides (and AMPs in general) as therapeutics to treat infectious diseases. In particular, the possibility of using biodegradable and biocompatible organic materials to encapsulate the peptide should be explored to give rise to new formulations for non- or less-invasive delivery routes (e.g., nasal, buccal/sublingual, or transdermal routes).

A second drawback that has slowed down the development of AMPs as new antimicrobial drugs is associated with the costs of large-scale production, which are generally much higher than those of small antibiotic molecules. Peptide compounds can be produced using a variety of techniques, including chemical synthesis, cell-free expression systems, recombinant DNA technologies for the production in heterologous cell systems, and transgenic organisms. Since natural Cecs and Cec-analogs generally show a low molecular weight (<4 KDa), chemical synthesis appears to be the best option for their production [145]. In addition, this technology allows the substitution of natural amino acids with atypical residues such as D-enantiomers, or the introduction of aa modifications (as in C-terminal amidation), often required in natural Cec and Cec-analog peptides. Chemical synthesis is undoubtedly an expensive approach [143]; however, due to the continuous development of efficient synthesis methodologies, progressive cost reductions for reagents, and competition among companies [6,145], considerable cost reductions are expected in the future. Consideration should also be given to the cost related to the development of possible AMP-based therapies compared to the social and economic burden caused by the current progressive and alarming spread of MDR infectious diseases [1]. Highlighting the USA as an example, 23,000 Americans are estimated to die annually with antibiotic resistant infections, while in 2018, direct national costs of treating antibiotic resistant infections have been projected to exceed $2 billion annually [146]. To these costs, other indirect economic and social costs should be added.

## 8. Conclusions

Insect Cecs and Cec-analog peptides are a class of AMPs that appear to be promising candidates as antibacterial therapeutics. These AMPs, tested alone or in combination with conventional antibiotics, show powerful antimicrobial activity against several important human pathogens, including MDR bacterial strains. They also exhibit low toxicity against mammalian cells and anti-inflammatory activity. Preliminary indications suggest that the development of new resistance phenomena against these peptides appears unlikely. However, few preclinical and no clinical analyses have been performed to date. In particular, long-term and/or longitudinal studies exploring potential side effects such as allergenicity or immunogenicity should be completed [6].

The intrinsic nature of Cec peptides, which makes them sensitive to protease degradation, together with the cost of large-scale production has slowed down or even impeded the development of Cec-based antimicrobial drugs. However, the advance of new strategies such as nanotechnologies will considerably reduce these limitations. The use of natural Cecs might allow the production of formulations active against Gram-negative bacteria, while the employment of Cec-analogs might give rise to therapeutics with a wide spectrum, effective against both Gram-negative and Gram-positive pathogens. In addition, given their anti-biofilm activity, Cecs and Cec-analogs might be used to coat biomaterials for medical devices as a strategy to prevent biomaterial-associated infections. Although further research and development studies are required, several lines of evidence suggest that both insect Cecs and Cec-analogs represent a suitable tool to counteract the alarming global spread of MDR pathogens.

## Figures and Tables

**Table 1:** In vitro antimicrobial activity and toxicity against mammalian cells of natural Cecropins (Cecs) and Cec-like peptides.

Insect	Species	Active Peptide (aa)	Antimicrobial Activity			Peptide conc. (μM)	
Order			Virus	Bacteria	Fungi	Cytotox.	Hem Act.
*Coleoptera*	*Oxysternon conspicillatum*	Oxysterlin 1 (39) [19]	-	G+, G−	weak	>28	>14
		Oxysterlin 2 (55) [19]	-	G−	NA	>19.75	>19.75
		Oxysterlin 3 (39) [19]	-	G−	NA	>28	>28
	*Acalolepta luxuriosa*	Cec (35) [20]	-	*M. luteus*, *E. coli*	-	-	-
	*Paederus dermatitis*	Sarcotoxin Pd (34) [21]	-	G+, G−	weak	-	16
*Diptera*	*Simulium bannaense*	SibaCec (35) [22]	-	G+, G−	-	58	58
	*Anopheles gambiae*	AngCec A (35) [23]	-	G+, G−	A	-	-
	*Aedes aegypti*	AeaeCec 1 (34) [24,25,26]	-	G+, G−	A	50 [26]	50 [26]
		AeaeCec 2–4 (34) [26]	-	-	-	50	50
		AeaeCec 5 (34) [26]	-	-	-	12.5	12.5
	*Aedes albopictus*	Cec A1 (35) [27,28]	-	*E. coli*, *Francisella*	-	-	-
		Cec B (35) [28]	-	*Francisella*	-	-	-
	*Culex pipens*	Cec A (34) [28]	-	*Francisella*	-	-	-
		Cec B2 (34) [28]	-	*Francisella*	-	-	-
	*Tabanus yao*	Cec TY1 (41) [29]	-	*B. subtilis S. aureus E. coli*	A	-	-
	*Hermetia illucens*	CLP1 (45) [30]	-	G−	-	-	-
	*Drosophila melanogaster*	Cec A (34) [23,24,31,32]	-	G+, G−	A	-	-
		Cec B (34) [31,32]	-	G−	A	-	-
	*Musca domestica*	Mdc (40) [33,34,35]	-	G+, G−	-	-	-
	*Glossina morsitans*	Cec (39) [36]	-	*M. luteus*, *E. coli*	-	-	-
	*Stomoxys calcitrans*	Stomoxyn (42) [37]	-	G+, G−	A	-	>10
	*Sarcophaga peregrina*	Sarcotoxins I A, B, C (39) [38,39,40]	-	G+, G−	-	-	-
	*Lucilia sericata*	Lser Cecs 1–6 (40) [41]	-	G−	NA	-	-
		LSerStomox1 (43) [41]	-	G−	NA	-	-
		LSerStomox 2 (42) [41]	-	G−	NA	-	-
*Lepidoptera*	*Hyalophora cecropia*	Cec A (37) [8,9,10,42,43,44,45]	HIV	G+, G−	A	[44,45]	100 [45]
		Cec B (35) [9,42,44,46]	-	G+, G−	A	30 [44]	500 [46]
		Cec D (36) [9,47]	PRRSV	G+, G−	-	-	-
	*Antheraea pernyi*	Cec B (35) [48,49]	-	G+, G−		25 [49]	200 [49]
		Cec D (36) [48]	-	G+, G−	-	-	-
		ApCec (38) [50]	-	*B. subtilis*, *E. coli*	-	-	62.5
	*Bombyx mori*	Cec A (35) [51,52]	-	G+, G−	A	-	-
		Cec B (35) [51,53]	-	G+, G−	NA	200 [53]	200 [53]
		Cec D (36) [51]	-	G+, G−	-	-	-
		Cec E (?) [51]	-	*B. thuringiensis*, G−	-	-	-
	*Galleria mellonella*	Cec D (39) [54,55]	-	*L. monocytogenes*	-	-	>115 [55]
	*Papilio xuthus*	Papiliocin (38) [56,57,58]	-	G+, G−	A	12.5 [58]	100 [58]
	*Spodoptera litura*	Spodopsin Ia (35) [59]	-	G+, G−	NA	-	-
		Spodopsin Ib (35) [59]	-	G+, G−	NA	-	-
		Cec A (35) [60]	-	G+, G−	-	-	-
		Cec B (35) [60]	-	G+, G−	-	-	-
	*Helicoverpa armigera*	Cec D (42) [61]	-	G+, G−	-	-	-
	*Heliothis virescens*	Cec B (35) [62]	-	*E. coli*	-	-	-
	*Agrius convolvuli*	AcCec D 1-3 (38) [63,64]	-	G+, G−	-	-	-
	*Artogeia rapa*	Hinnavin I (40) [65]	-	G+, G−	A	-	-
	(*Pieris rapae*)	Hinnavin II (38) [66]	-	G+, G−	A	-	-
	*Danaus plexippus*	DAN1 (37) [67]	-	G+ (weak), G−	-	-	49.56
		DAN2 (37) [67]	-	G+ (weak), G−	weak	-	48.97

Peptide conc. (µM): Peptide concentration showing no or weak toxicity in mammalian cells. Cytotox.: Cytotoxicity; Hem act.: Hemolytic activity against mammalian red blood cells; G+ Gram-positive bacteria; G−: Gram-negative bacteria; A: Active against tested species; NA: Not active against the tested species; -: Not determined; (?): Undetermined aa length.

**Table 2:** In vitro antimicrobial activity and toxicity against mammalian cells of Cec-analogs.

Peptide (aa)	Source	Modification	Antimicrobial Activity				Peptide Conc. (μM)	
			Virus	Bacteria	Fungi	Protozoa	Cytotox.	Hem Act.
SB-37 (38) [92]	*H. cecropia* Cec B	aa add./sub.	-	-	-	*P. falciparum*, *T. cruzi*	-	-
Shiva-1 (38) [46,92]	*H. cecropia* Cec B	aa add./sub.	-	G+, G−	NA	*P. falciparum*, *T. cruzi*	-	-
D-Cec B (35) [93]	*A. pernyi* Cec B	D-enantiomer	-	-	A	-	-	-
CecDH (32) [49]	*A. pernyi* Cec B	aa del.	-	G+, G−	-	-	25	100
ΔM1 (39) [55]	*G melonella* Cec D	N-term aa sub.	-	*Sa* (weak), *Ec*, *Pa*	-	-	-	115
ΔM2 (39) [55]	*G melonella* Cec D	N-term aa sub.	-	*Sa*, *Ec*, *Pa*	-	-	-	~60
Mdc–hly (?) [34]	*M. domestica* Mdc; human Lysozyme	Hybrid	-	G+, G−	-	-	-	-
CAMs (≤26) [94,95,96,97,98]	*H. cecropia* Cec A; *A. mellifera* Mellitin	Hybrids	-	G+, G−	A	*Plasmodium*	9 [96]	[98]
Ac-CAMs (15) [99]	*H. cecropia* Cec A; *A. mellifera* Mellitin	N-term fatty acid acylation	-	*Sa*, *Ec*, *Ab*	-	*L. pifanoi*	-	-
CAM-W (26) [98]	*H. cecropia* Cec A; *A. mellifera* Mellitin	aa sub.	-	G+, G−	A	-	-	3.12
CA-MAs (≤20) [100,101,102,103,104,105]	*H-cecropia* Cec A; *X. laevis* Magainin 2	Hybrids with aa sub.	virus–cell fusion inhibition	G+, G−	A	-	[105]	[105]
CA-LL37 (22) [106]	*H-cecropia* Cec A; human LL37	Hybrid	-	G+, G−	-	-	-	[106]
CecXJ-37C (37) [107]	*B. mori* Cec B	C-term aa add.	-	G+, G−	-	-	20	19
CecXJ-37N (37) [107]	*B. mori* Cec B	C-term aa add.	-	G+, G−	-	-	20	33

Peptide conc. (µM): Peptide concentration showing no or weak toxicity in mammalian cells; Cytotox.: Cytotoxicity; Hem act.: Hemolytic activity; sub.: Substitution; add.: Addition; del: Deletion; G+ Gram-positive bacteria; G−: Gram-negative bacteria; *Sa*: *S. aureus*; *Ec: E. coli*; *Pa: P. aeruginosa*; *Ab: A. baumannii*; A: Active against tested species; NA: Not active against the tested species; -: Not determined; (?): Not reported.
